# Cortical, Corticospinal, and Reticulospinal Contributions to Strength Training

**DOI:** 10.1523/JNEUROSCI.1923-19.2020

**Published:** 2020-07-22

**Authors:** Isabel S. Glover, Stuart N. Baker

**Affiliations:** Institute of Neuroscience, Newcastle University, Newcastle upon Tyne, NE2 4HH, United Kingdom

**Keywords:** corticospinal, reticulospinal, strength training

## Abstract

Following a program of resistance training, there are neural and muscular contributions to the gain in strength. Here, we measured changes in important central motor pathways during strength training in 2 female macaque monkeys. Animals were trained to pull a handle with one arm; weights could be added to increase load. On each day, motor-evoked potentials in upper limb muscles were first measured after stimulation of the primary motor cortex (M1), corticospinal tract (CST), and reticulospinal tract (RST). Monkeys then completed 50 trials with weights progressively increased over 8-9 weeks (final weight ∼6 kg, close to the animal's body weight). Muscle responses to M1 and RST stimulation increased during strength training; there were no increases in CST responses. Changes persisted during a 2 week washout period without weights. After a further 3 months of strength training, an experiment under anesthesia mapped potential responses to CST and RST stimulation in the cervical enlargement of the spinal cord. We distinguished the early axonal volley and later spinal synaptic field potentials, and used the slope of the relationship between these at different stimulus intensities as a measure of spinal input-output gain. Spinal gain was increased on the trained compared with the untrained side of the cord within the intermediate zone and motor nuclei for RST, but not CST, stimulation. We conclude that neural adaptations to strength training involve adaptations in the RST, as well as intracortical circuits within M1. By contrast, there appears to be little contribution from the CST.

**SIGNIFICANCE STATEMENT** We provide the first report of a strength training intervention in nonhuman primates. Our results indicate that strength training is associated with neural adaptations in intracortical and reticulospinal circuits, whereas corticospinal and motoneuronal adaptations are not dominant factors.

## Introduction

When subjects undertake a program of resistance exercise, they gradually grow stronger, becoming capable of increased levels of maximum voluntary contraction. The initial stages of strength training are dominated by neural adaptations rather than intramuscular mechanisms (Moritani and deVries, 1979; Sale, 1988; Folland and Williams, 2007). There is much evidence supporting this, including the absence of hypertrophy in the first few weeks of a strength training program (Komi, 1986; Jones and Rutherford, 1987; Akima et al., 1999), and the effect of cross-education in which unilateral training elicits bilateral gains (Enoka, 1988; Zhou, 2000; Lee and Carroll, 2007). Over the last few decades, attempts have been made to characterize these neural adaptations by examining elements of the corticospinal tract (CST), the dominant descending pathway in primates (Lemon, 2008). A recent meta-analysis proposed that strength training is characterized by changes in intracortical and corticospinal inhibitory networks, rather than corticospinal excitability (Kidgell et al., 2017). Adaptations may also occur at the level of the motoneuron, although there are technical limitations associated with these studies (Carroll et al., 2011).

Increasing evidence suggests that the reticulospinal tract (RST) plays an important role in primate upper limb function (Baker, 2011). In addition to its established role in postural control (Prentice and Drew, 2001; Schepens and Drew, 2004, 2006), the RST has been shown to project to motoneurons innervating both distal and proximal muscles (Davidson and Buford, 2004, 2006; Riddle et al., 2009) and contributes to motor control throughout the upper limb (Carlsen et al., 2012; Honeycutt et al., 2013; Dean and Baker, 2017). The bilateral nature of the RST (Jankowska et al., 2003; Schepens and Drew, 2006; Davidson et al., 2007), in combination with the synergies that result from its high degree of convergence (Peterson et al., 1975; Matsuyama et al., 1997; Zaaimi et al., 2018a), positions this pathway as a strong contender for the neural substrate of strength training. However, the RST has been largely overlooked in the strength training literature.

In support of this hypothesis, Lawrence and Kuypers (1968) reported an increase in strength 4-6 weeks after bilateral pyramidal tract (PT) lesions in monkeys, suggesting that strength gains can be achieved in the absence of the CST. Similarly, it has been suggested that an extrapyramidal pathway mediates recovery of strength after stroke (Xu et al., 2017). Given the adaptive changes that occur in the RST after corticospinal lesions (Zaaimi et al., 2012, 2018b), reticulospinal pathways are a likely candidate in mediating such strength adaptations.

The aim of this study was to compare the relative contributions of intracortical, corticospinal, and reticulospinal networks to the neural adaptations associated with strength training. We undertook two sets of experiments in rhesus macaques that were trained to perform a weightlifting task. First, we measured motor-evoked potentials (MEPs) in response to primary motor cortex (M1), PT, and medial longitudinal fasciculus (MLF) stimulation to assess adaptations in the cortex, CST and RST, respectively. Second, after completion of the strength training protocol, we measured spinal field potentials elicited with PT and reticular formation (RF) stimulation to assess spinal adaptations. To our knowledge, this is the first attempt to perform a strength training study in nonhuman primates and to investigate specifically strength-induced changes in reticulospinal function. Our results suggest that both intracortical and reticulospinal mechanisms contribute to the neural adaptations associated with strength training.

## Materials and Methods

All animal procedures were performed under United Kingdom Home Office regulationsin accordance with the Animals (Scientific Procedures) Act (1986) and were approved by the Animal Welfare and Research Ethics Board of Newcastle University. Recordings were made from 2 chronically implanted rhesus macaques (Monkeys N and L; 5.9-6.9 kg; both female). Both animals were intact before the study, with the exception of Monkey N who had lost parts of two fingers on the right hand in an unrelated incident.

### 

#### 

##### Behavioral task

**Figure 1.**
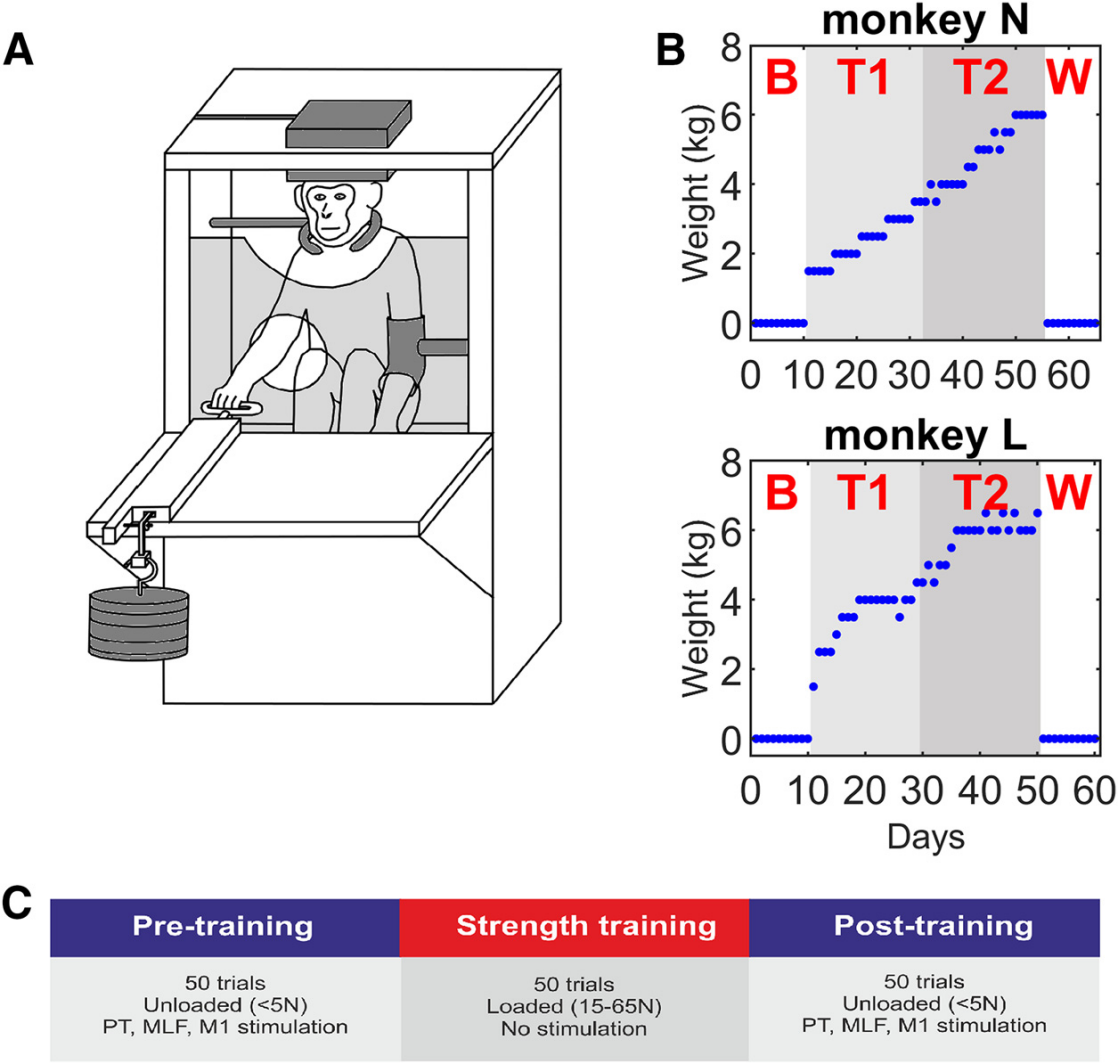
Strength training task. ***A***, Schematic of the experimental setup. The animal was atraumatically head-fixed, and wore a neck collar and a restraint on the left (untrained) arm. The right (trained) arm was free to reach through a hole in the front of the cage to pull a handle. The load was adjusted by adding weights to the other end of the handle. EMG activity was recorded and stimulation delivered via connectors on the headpiece. ***B***, Daily weight progression for each animal. The intervention consisted of four stages: a baseline period with no added load (***B***), strength training with low loads (T1), strength training with high loads (T2), and a washout period with no added load (W). Training was performed 5 d per week. ***C***, Training was performed 5 times per week. Each day began with a pretraining stimulation session in which the animals performed 50 unloaded trials while receiving PT, MLF, and M1 stimulation. This was followed by 50 loaded trials without stimulation for the strength training session. Finally, a second stimulation session was performed.

Both monkeys were trained to pull a loaded handle toward the body using their right hand. After each trial, the handle returned to its original position by the action of the load. Using a pulley system, weights could be attached to the handle so that the force required to pull it ranged from <5N in the unloaded control condition to 65N in the maximally loaded condition (Fig. 1). The task was self-paced, with the only time constraint being a minimum intertrial interval of 1 s. Trials were identified as successful if the handle was moved at least 4 cm; these were rewarded with food, and in the case of Monkey L, stimulation of the NAc as described below. Both monkeys were trained on the task in the unloaded condition before surgery.

##### Surgical preparation

Following successful training on the behavioral task, each animal underwent two surgeries: the first to implant a headpiece and EMG electrodes; and the second to implant cortical epidural electrodes and chronic stimulating electrodes in the PT and MLF. Both surgeries were performed under general anesthesia with full aseptic techniques.

The animals were initially sedated with an intramuscular injection of ketamine (10 mg kg^−1^). Anesthesia was induced with intravenous propofol (4 mg kg^−1^) and following intubation and insertion of a venous line, maintained through inhalation of sevoflurane (2%-3%) and continuous intravenous infusion of alfentanil (12 µg kg^−1 h−1^). During surgery, hydration levels were maintained with a Hartmann's solution infusion, a thermostatically controlled heating blanket maintained body temperature, and a positive pressure ventilator ensured adequate ventilation. Pulse oximetry, heart rate, blood pressure, core and peripheral temperature, and end-tidal CO_2_ were monitored throughout surgery. Anesthetic doses were adjusted as necessary during surgery and a full program of postoperative analgesia and antibiotic care followed surgery.

In the first surgery, a headpiece was implanted to enable atraumatic head fixation during the behavioral task and to provide a mount for the electrode connectors. The headpieces were designed to fit the bone surface using a structural MRI scan, 3D printed with titanium powder, coated with hydroxyapatite, and surgically attached to the skull using the expanding bolt assemblies described by Lemon (1984). During the same surgery, electrodes for EMG recording were bilaterally implanted into the first dorsal interosseous, flexor digitorum superficialis (FDS), flexor carpi radialis (FCR), extensor digitorum communis (EDC), biceps brachii, triceps brachii, pectoralis major, and posterior deltoid muscles. Electrodes were placed bilaterally with the exception of the FCR, which was implanted on the left side of Monkey L and right side of Monkey N. Each EMG electrode was custom-made and consisted of a pair of insulated steel wires (AS632, Cooner Wire), bared for 1-2 mm at their tips, which were sewn into the muscles using silk sutures. The wires were tunneled subcutaneously to the headpiece on which their connectors were mounted.

In a second surgery, performed 3 weeks later, two custom-made electrodes (75 μm stainless-steel wire insulated with Teflon, bared for ∼1 mm at the tip; FE6321, Advent Research Materials) were implanted onto the dural surface above each M1 to allow stimulation of the motor cortex. One electrode was placed medial, and one lateral, over the upper limb representation as judged by ML stereotaxic coordinate (∼12 mm lateral to the midline); connectors were cemented onto the headpiece using dental acrylic. Four parylene-insulated tungsten electrodes (LF501G, Microprobe) were chronically implanted bilaterally into the medullary PT and MLF, rostral to the pyramid decussation, to allow stimulation of the CST and RST, respectively. The double-angle stereotaxic technique, described by Soteropoulos and Baker (2006), was used to aim each electrode at the desired target, from a craniotomy placed at an arbitrary convenient location on the headpiece. The optimal position for the PT electrodes was defined as the site with the lowest threshold for generating an antidromic cortical volley in ipsilateral M1, without eliciting a contralateral M1 volley at 300 µA. The optimal MLF electrode position was defined as the site ∼6 mm above the PT electrode, which had the lowest threshold for generating a spinal volley without an antidromic cortical volley. All electrodes targeted an AP coordinate at the interaural line (AP0). The DV location of the electrodes was estimated as 6.5–9.3 mm below the interaural line for PT, and 0.4 above to 5.5 mm below for MLF. The threshold for evoking a spinal volley was 10–20 µA for PT and 20–100 µA for MLF. Cortical volleys were obtained by recording from the cortical electrodes implanted at the start of the surgery. Spinal volleys were recorded using a wire temporarily positioned in the paraspinal muscle near the cord with a needle; this was removed at the end of surgery.

Monkey L underwent an additional surgery before the start of the strength training protocol to implant an electrode into the NAc, stimulation of which has been shown to be an effective behavioral reward (Bichot et al., 2011). Following sedation with ketamine (10 mg kg^−1^), a burr hole was drilled above the target penetration site and sealed with a thin layer of acrylic. The following day, in the awake head-fixed animal, the acrylic was removed and an insulated tungsten electrode was driven toward the NAc target location. To optimize position, stimulus trains were given through the electrode as it was advanced in 0.5–1 mm steps (1.0 mA biphasic pulses, 0.2 ms per phase, 200 Hz frequency, 200 ms train duration), and the facial expressions and vocalizations of the animal monitored until an optimal response were observed. Typically, we found a sequence as the electrode was advanced: the animal first showed a mild orienting reaction following the stimulus, with characteristic retraction of the ears. Further electrode advancement produced vocalization (typically grunting), which became stronger at deeper sites. At the optimal site, vocalization could be produced at a threshold of 100 µA. The electrode was then fixed in place with dental acrylic, sealing the burr hole, and a connector cemented onto the headpiece with dental acrylic. During subsequent training sessions, Monkey L received NAc stimulation every 1–3 successful trials at random, with the stimulation intensity increased as necessary to maintain motivation (1.0–2.5 mA biphasic pulses, 0.2 ms per phase, 200 Hz frequency, 200 ms train duration).

##### Experiment 1: EMG recordings

Following recovery from surgery and refamiliarization with the task, the animals underwent 12 week (Monkey L) and 13 week (Monkey N) strength training protocols. The following was performed 5 d per week. Each day began with an initial stimulation session in which the animals performed 50 unloaded trials of the task while receiving stimulation of the four brainstem electrodes (bilateral PT and MLF: 500 µA biphasic pulses, 0.2 ms per phase, 2 Hz repetition rate) and four cortical electrodes (bilateral medial and lateral M1: 3 mA biphasic pulses, 0.2 ms per phase, 2 Hz repetition rate) in pseudo-random order. The unloaded task served to generate low-level background EMG activity on which MEPs could be recorded. The animals then performed the strength training session consisting of 50 loaded trials (1.5–6.5 kg); no stimulation was delivered during this session. Finally, to assess short-term adaptations, a second stimulation session was performed with the same format as the first. These three daily sessions will subsequently be referred to as the pretraining, strength training, and post-training sessions (Fig. 1*C*).

During all of these sessions, the task was performed with the right arm while the left arm was held in a restraint, a collar placed around the neck, and the head atraumatically fixed by the headpiece to allow connection to the EMG and stimulating electrodes (Fig. 1*A*). EMG (5 kHz sampling rate, 200-1000 gain, 0.1 Hz to 10 kHz bandpass) and task parameters, such as lever position and stimulus times, were stored to disk. The total training each day took ∼20 min.

The first 2 weeks (baseline period) and last 2 weeks (washout period) of the training protocol were performed without weights during the strength training session to establish an unloaded baseline measure and to assess post-training washout effects. During the remaining 8-9 weeks, the weights were gradually increased day by day, as tolerated by the animals (Fig. 1*B*).

All analyses of EMG data were performed offline using custom software written in MATLAB. EMG recordings were high pass filtered at 30 Hz and then full-wave rectified. Background EMG activity was measured over a 40 ms window (from 50 to 10 ms before each stimulus) for each stimulus trial. Single-stimulus trials were only included in the analysis if they generated a measurable response, defined as exceeding background EMG activity for a continuous period of at least 3 ms, measured 5–25 ms after stimulus delivery. Only stimulus-muscle combinations, which generated reliable MEPs, were included in the subsequent analyses. These were defined as follows. First, to test whether there was a measurable response, mean sweeps were calculated for the 10 d baseline period and for the 10 d washout period. The stimulus-muscle pair were only included if both of these values exceeded a mean background EMG for a continuous period of at least 5 ms. Second, to test the stability of the MEP, correlation coefficients were calculated between the mean stimulus-response sweeps of the first 5 d and second 5 d of the baseline period. Stimulus-muscle pairs were only included if *R*^2^ > 0.75 and *p* < 0.05. If the stimulus-muscle pair met both these criteria, it was concluded that a MEP was reliably present throughout the experimental period (from baseline to washout), and that without intervention (during the baseline period), it was consistent. MEP amplitude was then quantified as area under the curve above background EMG between cursors. These cursors were set to the onset and offset of response above background EMG determined from the averages in the baseline period.

Because of the variation in background EMG activity, and the known effect of this on MEP amplitude (Hess et al., 1987), MEPs were normalized by dividing by their corresponding background EMG measure. The human transcranial magnetic stimulation (TMS) and transcranial electrical stimulation (TES) literature suggests that a linear relationship does not exist between background EMG level and MEP size (Kischka et al., 1993; Taylor et al., 1997), but can instead plateau above a certain background EMG, depending on the muscle. Nonetheless, we have persisted with this normalization method because, although it may attenuate our effects by overcompensating for background EMG activity, it reduces the likelihood that any trends observed are simply due to changes in background.

To assess short-term effects of individual strength training sessions, the daily recording sessions were grouped into four weight ranges for each monkey: no weight (0 kg, unloaded task), light (0.5–3.5 kg), moderate (4.0–5.0 kg), and heavy (5.5–6.5 kg). Effects were expressed as apercentage change in MEP size from the pretraining session to the post-training session. Similar percentages were obtained for the different muscles, and so the results were grouped simply by averaging the percentage change values across all of the included muscles for each stimulus and day. Statistically significant (*p* < 0.05) changes in MEP size were identified with a one-sample *t* test, and multiple comparisons were corrected within each monkey using a Benjamini-Hochberg correction with a false discovery rate of 5%. This analysis was repeated for normalized MEPs and background EMG measures.

To assess long-term adaptations to strength training, the pretraining daily sessions were grouped into four stages for each monkey: baseline, strength training 1, strength training 2, and a washout period (Fig. 1*B*). These sessions are time-based in contrast to the sessions used for assessment of short-term training adaptation, which are weight-based. For single muscles, mean MEP size for each stage was expressed as a percentage of the mean baseline period MEP. To combine the responses across muscles to provide a single measure for each stimulus, the variance of the baseline period MEPs was determined for each muscle and used to calculate an inverse-variance weighted daily average (Hartung et al., 2008), so that the most emphasis was placed on the stimulus-muscle pairs, which had the most reliable baseline MEPs. These values were then averaged across days to produce a single value per stimulus and training stage. Independent *t* tests were performed relative to the baseline period, and multiple comparisons were corrected within each monkey using a Benjamini-Hochberg correction with a false discovery rateof 5%. Homogeneity of variance was assessed with Levene's test; Satterthwaite's approximation for the effective degrees of freedom was used when equal variance could not be assumed. This analysis wasperformed for both the original MEP values and background EMG-normalized values (see above). Similarly to the single-muscle MEPs, background EMG activity for each muscle was expressed as a percentage of the mean baseline period value.

##### Experiment 2: spinal recordings

Following completion of the 12 to 13 week strength training protocol, each animal continued with a daily strength training regimen as part of a separate study in which single-unit recordings were made from M1 and RF. Over a 3 month period, 20–50 trials were performed ∼5 d per week with each of the following weights: 0.5, 1, 1.5, 2, 3, 4, and 6 kg; hence, the animals received as least as much strength training as in the main intervention. An experiment under terminal anesthesia was then performed in which recordings were made from the spinal cord to assess changes in synaptic efficacy.

Initial sedation was achieved with an intramuscular injection of ketamine (10 mg kg^−1^). Anesthesia was then induced with intravenous propofol (4 mg kg^−1^) and maintained through intravenous alfentanil(24–27 µg kg^−1^ h^−1^) and inhalation of sevoflurane (3%). Pulse oximetry, heart rate, blood pressure (measured continually by a central arterial cannula), core and peripheral temperature, and end-tidal CO_2_ were monitored throughout surgery, and anesthetic doses adjusted as necessary to ensure deep general anesthesia was maintained.

A craniotomy and laminectomy were performed to expose the right motor cortex and cervical spinal cord, respectively. The vertebral column was clamped at the high thoracic and mid-lumbar levels and the head fixed in a stereotaxic frame, with the neck flexed by ∼60°. The anesthetic regimen was then switched to an intravenous infusion of alfentanil (24–67µg kg^−1^ h^−1^), ketamine (6–10 mg kg^−1^ h^−1^), and midazolam (0.3 mg kg^−1^ h^−1^), which we have found provides stable anesthesia while preserving good levels of excitability across the motor system.

Although stimulating electrodes were already implanted into the PT and MLF, new electrodes were inserted for use during the spinal recordings, as we were concerned that gliosis around the tips since implant was likely to reduce the efficacy of the chronic electrodes by variable and unknown amounts. As the MLF is a small structure, we targeted the stimulating electrodes for the terminal experiment to the nucleus gigantocellularis of the RF instead. Electrode implant used an approach through a craniotomy adjacent to the foramen magnum. This minimized the distance traveled and associated risk of deviation from the intended trajectory. Electrode placement was optimized with reference to cortical and spinal volleys recorded from epidural ball electrodes. Penetrations were made at an angle of 30° relative to the spinal cord. Each electrode was first zeroed to the obex landmark on the brainstem. To target the PT, penetrations were made 1 mm lateral and 2 mm caudal to obex; electrodes were fixed 7.7–9.4 mm below the depth of obex. To target the RF, penetrations were made 2 mm lateral and 2 mm rostral to obex; electrodes were fixed 4.3–5.5 mm below the depth measured at obex.

**Figure 2.**
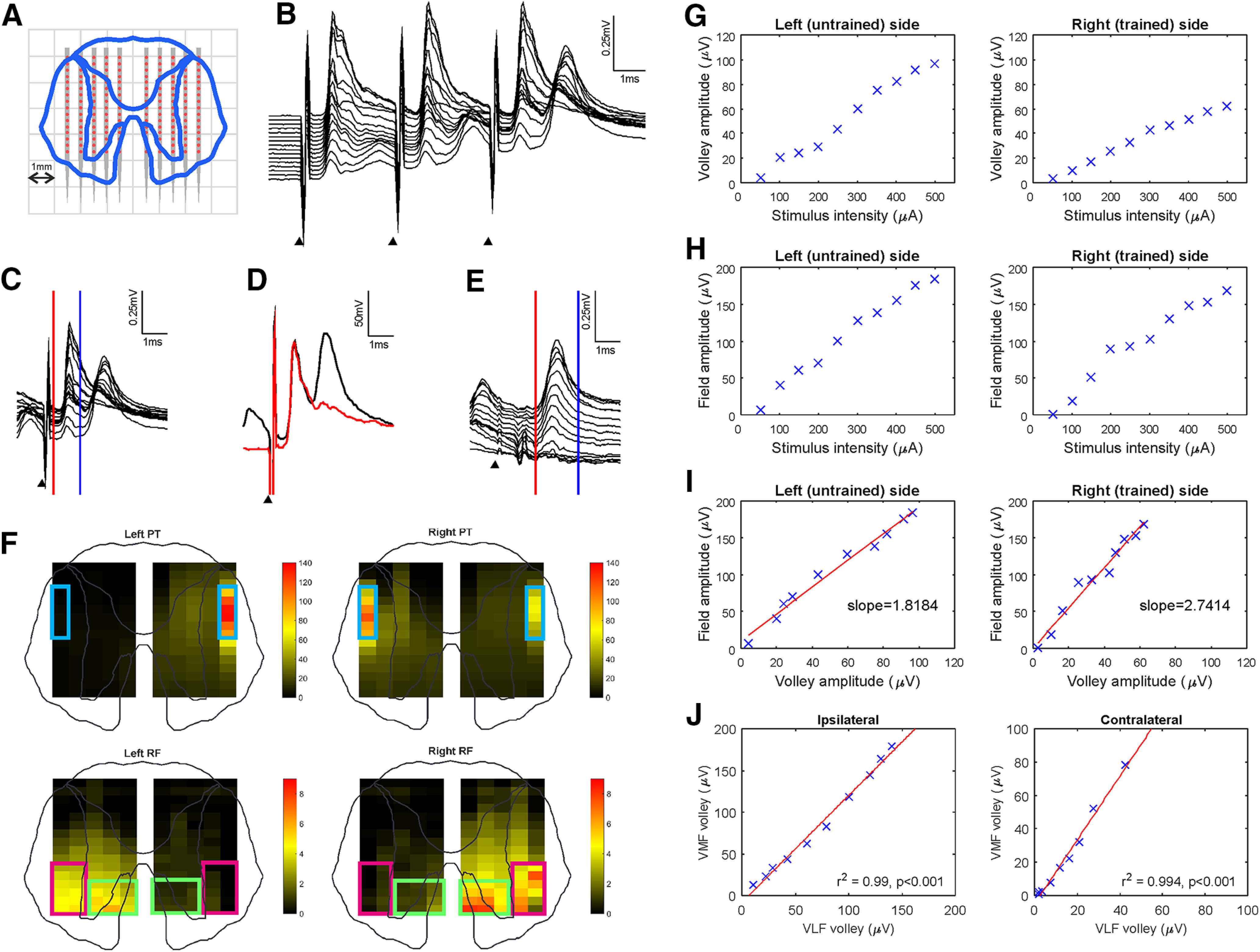
Spinal recording methods. ***A***, A single electrode was inserted into the spinal cord at 500 µm intervals relative to the midline and at a constant depth to produce a grid of recordings. The electrode consisted of 16 contacts (red dots) spaced 240 µm apart, with the first contact 1.5 mm from the tip. ***B-E***, Example spinal traces recorded from all contacts of a single electrode positioned 2 mm left of the midline at the caudal site of Monkey N in response to a 300 µA left PT stimulus. Black arrows indicate stimulus delivery. ***B***, Recording of response to a train of three stimuli. Note the constant size of the volley in contrast to the growing field. ***C***, The amplitude of the volley was measured as the maximum value between two cursors. ***D***, Example application of field isolation. The response to a single stimulus (red) was subtracted from the response to the last stimulus in a train of three (black), to isolate the field from the decay of the volley. ***E***, The amplitude of the isolated field was measured as the maximum value between two cursors. ***F***, Spinal volley amplitudes recorded with left PT, right PT, left RF, and right RF stimulation were used to define the DLF (blue squares), VLF (purple squares), and VMF (green squares) for their respective stimuli. The recordings shown are from the rostral site of Monkey L with a 200 µA stimulus intensity. ***G-J***, Example of gradient calculation for field and volley relationship. With data recorded from the deepest contact of the caudal electrode of Monkey N, 0.5 mm to the left (first column) and right (second column) of the midline, in response to contralateral PT stimulation with the volley assessed at the DLF. Volley (***G***) and field (***H***) amplitude were measured for a range of stimulus intensities. ***I***, For each stimulus intensity, field amplitude was plotted against volley amplitude. A linear regression was performed to calculate the gradient of this volley-field relationship, which gave a measure of the synaptic efficacy of the stimulus at that site in the cord. The difference between gradients for mirrored locations on the cord was calculated (e.g., 2.7414–1.8184 = 0.9230) to compare the effects of the unilateral strength training intervention. The significance of this difference was assessed with an ANCOVA (here *p* = 0.000125). This analysis was repeated for each position on the recording grid (***A***), for each recording site (rostral or caudal) and each monkey. ***J***, Correlation of volley amplitude for VLF and VMF. Example volley recordings made from sites corresponding to VLF and VMF for the left side of the cord at the caudal site of Monkey N in response to ipsilateral (left) and contralateral (right) RF stimulation. Each data point represents a different stimulus intensity. A significant correlation was observed between VLF and VMF volleys.*r*^2^ and *p* values are shown on each panel.

To record spinal field potentials, the dura was opened at a rostral (C5–C6) and caudal (C6–C7) site on the cord. Recordings were made using a single 16-channel electrode (LMA, 50 µm contacts spaced 240 µm apart, Microprobe) per site. A series of 10 penetrations was made, progressing from lateral to medial in 500 µm increments. Successive recordings alternated from the left to the right side of the cord, and vice versa, minimizing the likelihood of differences being observed between the two sides due to changes in excitability with time, as may occur with progressive changes in anesthetic dose. The 500 µm spacing of penetrations and 240 µm spacing between electrode contacts produced a grid of recording sites across a cross-section of the cord (Fig. 2*A*). For each penetration, an intensity series was delivered through each of the newly implanted PT and RF electrodes for both single stimuli (50–500 µA biphasic pulses in 50 µA increments, 0.2 ms per phase, 4 Hz repetition rate) and trains of three stimuli (50–500 µA biphasic pulses in 50 µA increments, 0.2 ms per phase, 4 Hz repetition rate, 333 Hz train frequency). In Monkey N, spinal field potential recordings were made under neuromuscular blockade (atracurium; 0.75 mg kg^−1^ h^−1^ i.v.); no neuromuscular block was used in Monkey L. The spinal recordings (25 kHz sampling rate) and stimulation parameters were stored to disk.

The aim of these recordings was to assess whether there were changes in the spinal responses to stimulation on one side of the cord relative to the other as a result of strength training the right arm. We could identify two components in our recordings (Fig. 2*B*). The earliest component was a volley, generated by axons in the stimulated descending tract; this represents the input to the cord. This followed multiple stimuli faithfully, and was present even for weak stimuli. A later component represented the response of spinal circuits to the descending input. The field potentials were small, even with the highest intensity stimuli following single shocks but grew with trains of three stimuli (Fig. 2*B*). In intracellular recordings, we would normally consider such temporal facilitation as indicative of a disynaptic linkage (Witham et al., 2016), but the short latency of the field after the corresponding volley (<1 ms) is only compatible with a monosynaptic connection. We consider that the field represents mainly a spiking response in local neurons, which became more probable with successive stimuli in a train due to temporal summation. The location of the fields, which were concentrated within the ventral horn and intermediate zone, was compatible with the regions known to receive strong input from descending pathways.

The amplitude of the volley was measured as the difference between maximum and minimum voltage between cursors placed manually (Fig. 2*C*), using the response to a single shock of the train. To prevent contamination of the field potentials with the decay of the volley, the response evoked by a single stimulus, in which no field was present, was subtracted from the response after the third stimulus in a train to produce an isolated field (Fig. 2*D*). The amplitude of the field was then measured as the difference between maximum and minimum voltage in a window placed later after the stimulus than that used for the volley (Fig. 2*E*). Cursor positions were determined individually to be optimal for each monkey, recording site and stimulus.

Volley amplitude measurements for each penetration and electrode contact were used to generate surface plots representing cross-sections of the spinal cord (Fig. 2*F*). These contained clear spatial peaks, corresponding to the dorsolateral funiculus (DLF; Fig. 2*F*, blue boxes), activated by the PT stimuli; and the ventrolateral funiculus (VLF; Fig. 2*F*, red boxes) and ventromedial funiculus (VMF; Fig. 2*F*, green boxes), activated by the RF stimuli. The locations corresponding to these regions were manually selected for each monkey and each electrode (Fig. 2*F*), and the volley amplitudes across them was summed to give a measure of the total input to the cord by that stimulus for each stimulus intensity. For a given stimulus, the amplitude of these volleys could be plotted versus intensity (Fig. 2*G*).

For a given spinal location and stimulus, the field amplitude could also be plotted versus intensity yielding a recruitment curve (Fig. 2*H*). It would be possible to use this as a measure of the spinal response, but slight asymmetries between the placement of stimulating electrodes on the two sides could lead to inaccuracies. Instead, we chose to plot the field amplitude versus volley amplitude (Fig. 2*I*), as they both varied with stimulus intensity. This represents a true input-output curve for each location in the cord, where the input values were the summed volley amplitudes for each region of the white matter (DLF, VLF, and VMF) and the output values were field amplitudes at each spinal location. This relation was very close to linear; the slope of the regression line (Fig. 2*I*) represents the gain of the spinal circuits. We used this as our measure of synaptic efficacy. Comparing the slopes of the lines for corresponding locations mirrored across the midline thus gives a measure of changes in synaptic efficacy on one side of the cord compared with the other. The difference between the two gradients was calculated and an ANCOVA performed to test the significance of this. Positions with a negative gradient or an insignificant regression (*p* > 0.05) were excluded from subsequent analysis.

We had available recordings from a caudal and rostral level of the cervical spinal cord, in 2 monkeys. To summarize the results across these four recordings in a single image, the gradient differences between the two sides for each stimulus were normalized to scale between 0 and 1, and an average of the normalized gradient differences was calculated. The significance of group changes was assessed by assigning each of the original gradient differences: 0 for an insignificant change, 1 for a significantly steeper gradient on the right cord compared with the left, and −1 for a significantly shallower gradient on the right cord compared with the left. Summing these values across the four available recordings gave a score from −4 (all recordings showed a significantly shallower gradient on the right side of the cord) to 4 (all recordings showed a significantly steeper gradient on the right side of the cord). By simulating all possible combinations of scores across the 5 (penetrations) × 16 (electrode contacts) recording grid and assuming the null hypothesis that any differences arise by chance, we found that a score of ≥2, or ≤−2, could be considered significant at *p* < 0.005. This analysis was only performed for DLF and VLF recordings since we observed a highly significant correlation between VLF and VMF volley amplitude (Fig. 2*J*), presumably due to similar activation of these two reticular pathways by our RF stimulus.

##### Histology

After completion of the study, electrolytic lesions were made by passing current through the PT, MLF, and RF electrodes (100 µA for 20 s). Anesthesia was then increased to a lethal level, and animals were perfused through the heart with PBS followed by formal saline.

The brainstem and spinal cord were removed and immersed first in formalin and then in ascending concentrations of sucrose solution (10%, 20%, 30%) for cryoprotection. A freezing microtome was used to cut 80 µm sections, which were mounted and stained with cresyl violet to enable anatomic reconstruction of the brainstem stimulating electrode positions.

## Results

### Task performance

Both animals complied well with the task, completing the required 150 trials on all but a few days. The progression of weight added to the task during the strength training session differed between the 2 animals, and it is likely that the first few weeks of this (Training 1) constituted familiarization with lifting weight rather than intensive strength training. It was not possible to perform measures of maximum voluntary contraction; and so unlike in human strength training experiments, we were unable to fix the load to generate a certain percentage of maximum voluntary contraction. Instead, subjective assessments were made of each animal's capability, in terms of both strength and motivation, and the weights increased accordingly. By the end of the intervention, each monkey was performing 50 consecutive trials with at least 6 kg, which was approximately equivalent to their body weight. This would be sufficient to constitute a strength training program, based on the human literature (Schoenfeld et al., 2016).

**Figure 3.**
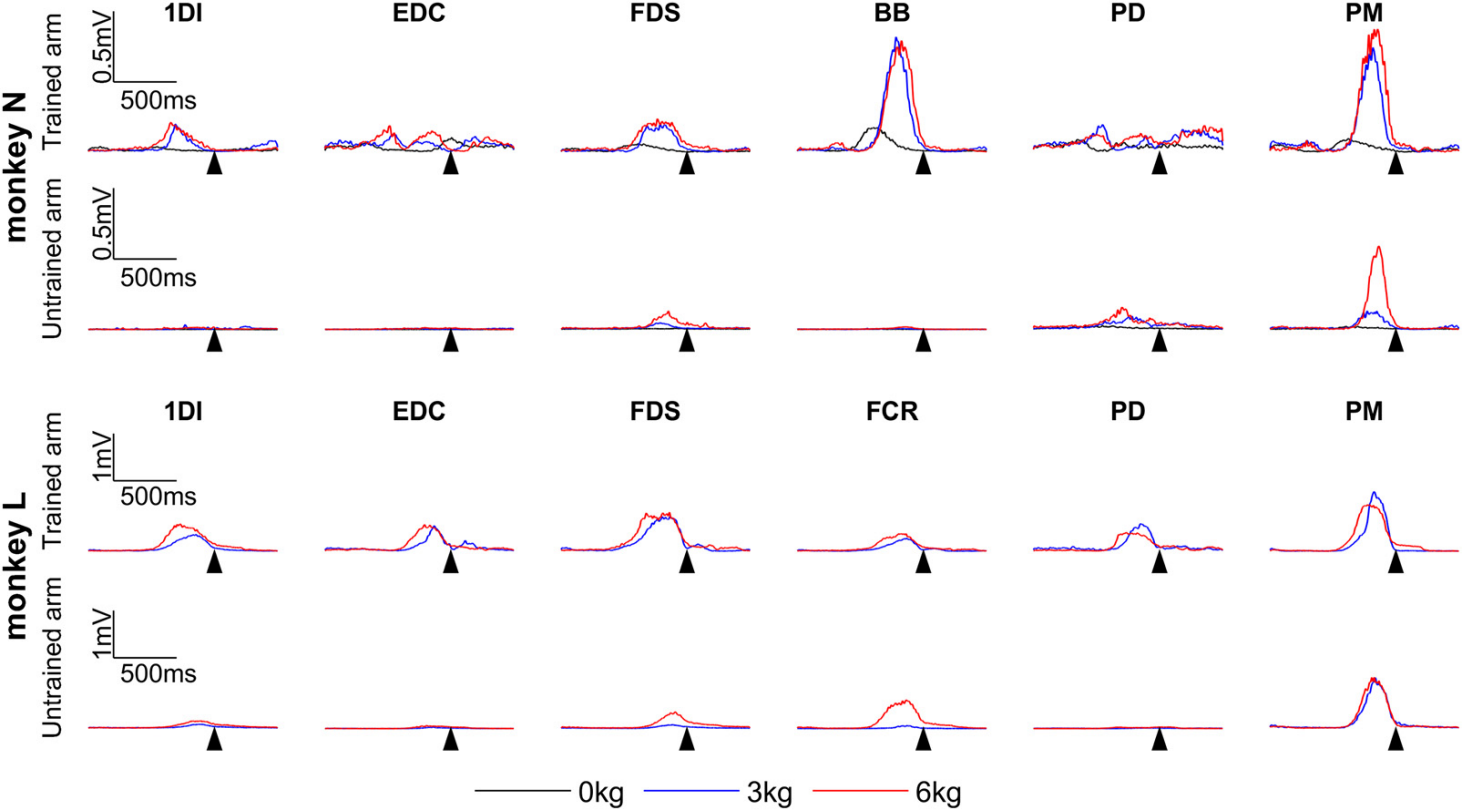
Example EMG activity during task with different loads. Mean rectified EMG activity for all trials (*n* = 50) on a single day recorded from muscles on the right (trained) arm and left (untrained) arm. Recordings are from the strength training sessions of day 2 (0 kg), day 26 (3 kg), and day 50 (6 kg) for Monkey N; and day 2 (0 kg), day 15 (3 kg), and day 36 (6 kg) for Monkey L. Sweeps are aligned to maximum lever displacement (arrow). The left arm was held in a restraint during these recordings. Columns relate to different muscles.

The task was found to activate all recorded muscles on the right (trained) arm (Fig. 3), with increasing muscle activation with load. Although designed to be unilateral, the task generated some bilateral activation, particularly in proximal muscles and with heavier loads (Fig. 3). Since the left (untrained) arm was held in a restraint, this activation does not represent bimanual task performance but instead may result from mirror activation (Armatas et al., 1994; Mayston et al., 1999; Ejaz et al., 2018) or postural bracing.

### MEP recordings

**Figure 4.**
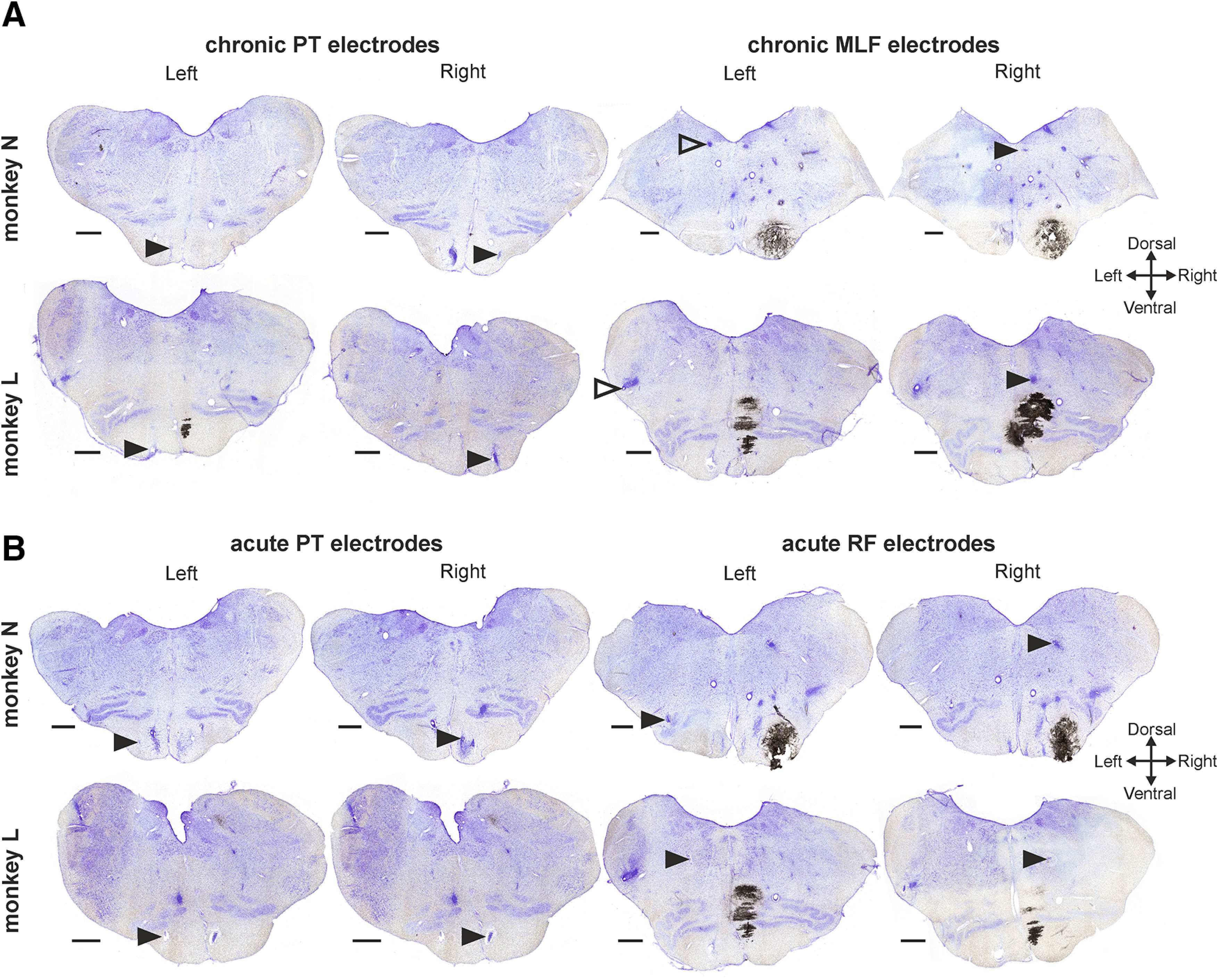
Histology confirmation of electrode locations. Cresyl violet-stained coronal sections for (***A***) chronic PT and MLF electrodes and (***B***) acute PT and RF electrodes for each monkey. Arrowheads indicate the location of the electrode tips. Solid black arrowheads indicate appropriately positioned electrodes. Empty arrowheads indicate the inappropriately positioned chronic left MLF electrodes in both monkeys (see Results). Scale bars, 1 mm.

MEPs were recorded in response to PT, MLF, and M1 stimulation. The position of the PT and MLF electrodes was verified histologically after completion of the study (Fig. 4). Although implanted bilaterally, the left MLF electrode was incorrectly positioned in both monkeys (Fig. 4) and did not reliably elicit MEPs; this has therefore been excluded from the analysis. In contrast, the right MLF electrode elicited clear MEPs bilaterally in both monkeys, and so, for the purposes of this analysis, has been used to assess reticulospinal output in a nonlateralized manner. It is likely that the bilateral effect of this electrode relates both to current spread across the midline and the established bilateral effects of the RST (Davidson and Buford, 2006).

**Figure 5.**
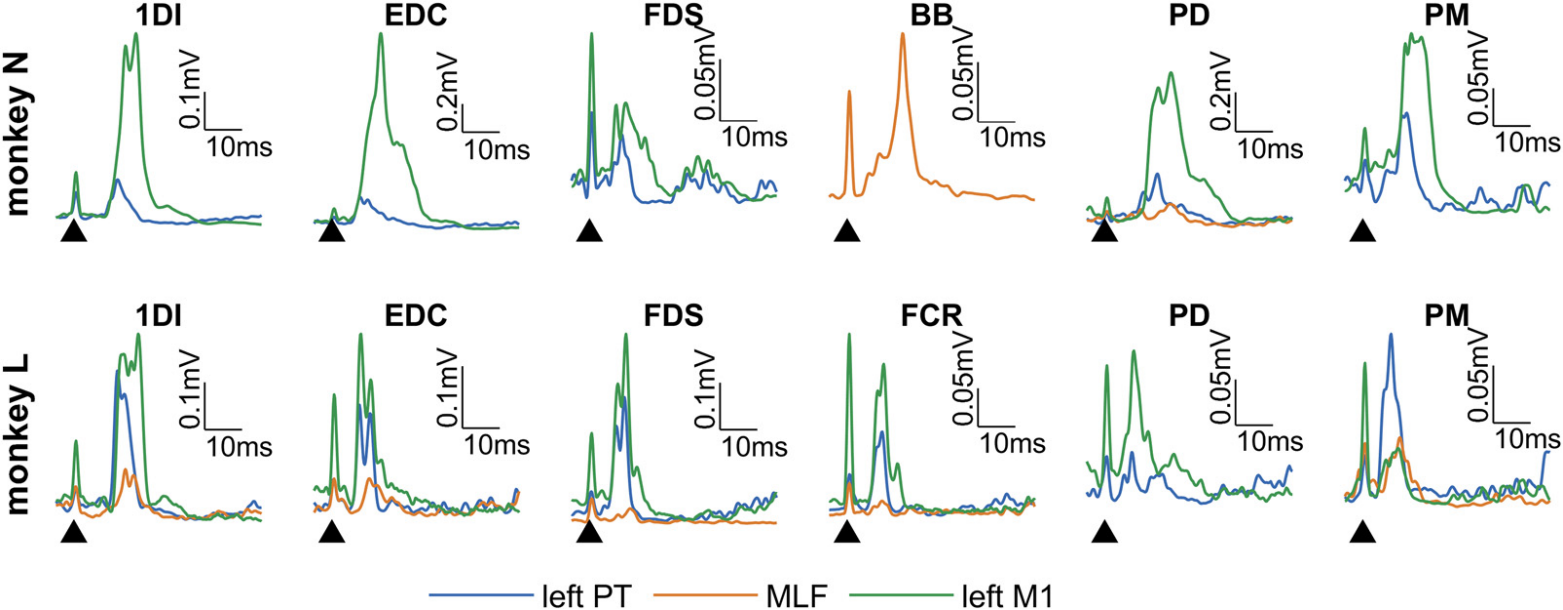
Example MEP recordings. Mean rectified EMG traces showing MEPs recorded from the muscles of the right (trained) arm during the last day of prestrength training stimulation during the baseline period (day 10). Only stimuli giving a clear MEP in the specified muscle are shown. Sweeps are aligned to the stimuli (arrows).

MEPs were consistently observed in most muscles in response to contralateral PT and cortical stimulation (Fig. 5). Similar results were observed with both the medial and lateral cortical electrodes, so only responses to the lateral cortical electrodes have been presented. Stimulus-muscle pairs that reliably generated MEPs were identified (see Materials and Methods). This analysis resulted in the omission of the EMG recordings from the left (untrained) arm since only 10 of a possible 36 muscle-stimulus pairs met the MEP inclusion criteria (data not shown).

Epidural electrical stimulation over the motor cortex generates D- and I-waves (Rosenthal et al., 1967; Di Lazzaro et al., 2004), implying that it can activate corticospinal cells directly and also via intracortical circuits. This is therefore a similar stimulus to TMS in humans. In contrast, the PT electrodes were positioned to stimulate the descending corticospinal fibers distant to the cortex, so that the volley evoked should be independent of cortical excitability. This stimulus can be considered comparable with cervicomedullary (or transmastoid) stimulation in humans, and to a lesser extent TES, both of which are thought to stimulate corticospinal axons directly (Rothwell et al., 1994; Taylor and Gandevia, 2004). Importantly, comparisons between M1 and PT MEPs can give an indication of whether adaptations are occurring within the cortex or subcortical levels, similarly to the comparison between TMS and TES or transmastoid stimulation in the human literature (Rothwell et al., 1994; Taylor and Gandevia, 2004). Although the MLF contains reticulospinal (Jankowska et al., 2003; Edgley et al., 2004), vestibulospinal (Nyberg-Hansen, 1964a; Wilson et al., 1968), and tectospinal fibers (Nyberg-Hansen, 1964b), we propose that the most important output from MLF stimulation is likely to be RST activation, for reasons discussed previously (Riddle et al., 2009; Riddle and Baker, 2010).

### Short-term training adaptations

**Figure 6.**
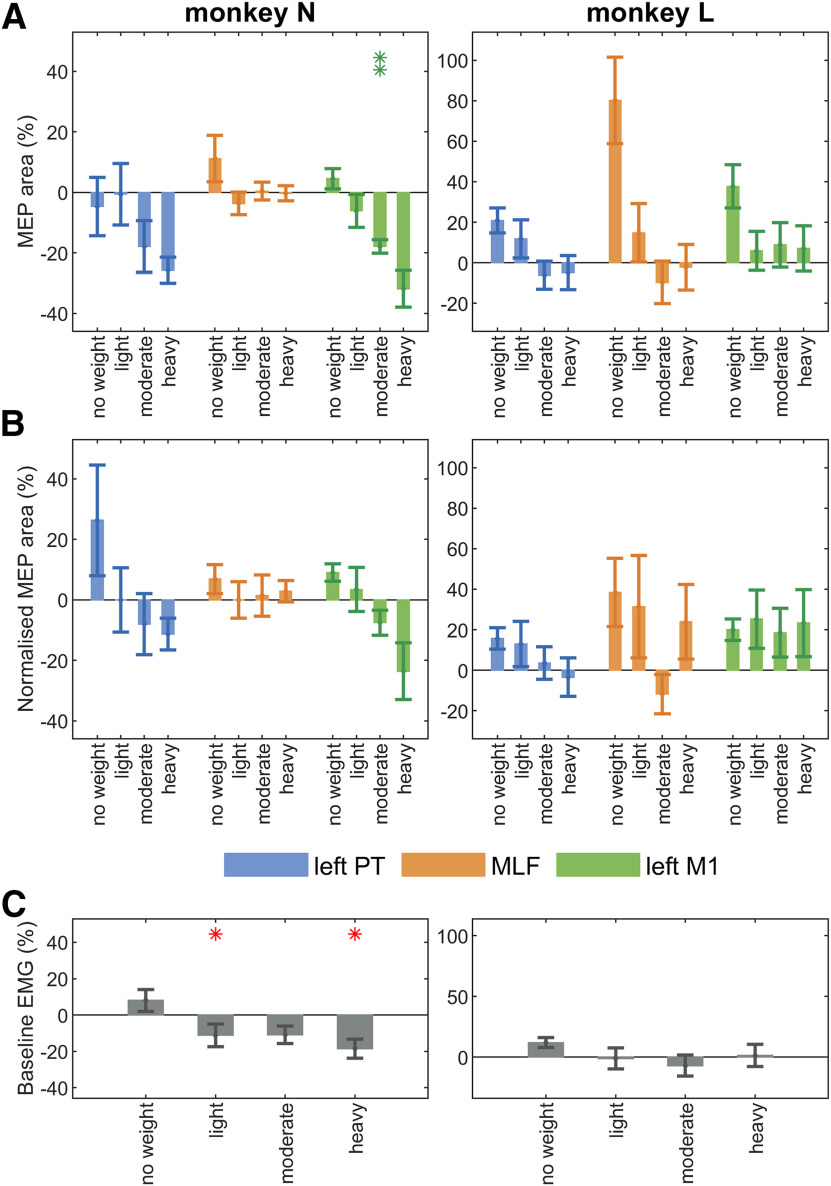
Short-term adaptations to strength training in the right (trained) arm. Percentage change from the prestrength training to the post-strength training stimulation session, summarized across all muscles for (***A***) original MEPs, (***B***) background-normalized MEPs, and (***C***) background EMG activity. MEP area was calculated as the area above background EMG for a custom window for each muscle-stimulus combination. Background EMG was calculated as mean rectified EMG activity measured over a 40 ms window (−50 to −10 ms) before each stimulus. Results have been averaged across all muscles on the right (trained) arm that showed a clear MEP for the given stimulus (see Fig. 5), and across all included muscles for background EMG activity. MEPs were grouped into weight ranges: no weight (baseline period), light (0.5-3.5 kg), moderate (4-5 kg), and heavy (5.5-6.5 kg). MEP percentage change values are statistically significant (*p < 0.05; **p < 0.01) from zero (no change in MEP size), as identified with one-sample *t* tests. Multiple comparisons were corrected within each monkey using a Benjamini-Hochberg correction with a false discovery rate of 5%. Degrees of freedom (no weight, light, moderate, heavy) for original and background-normalized MEP *t* tests for Monkey N: left PT (9, 17, 8, 15), MLF (9, 15, 8, 5), left M1 (9, 19, 7, 6); and Monkey L: left PT (6, 7, 13, 14), MLF (6, 7, 13, 14), left M1 (6, 5, 12, 14). Degrees of freedom (no weight, light, moderate, heavy) for background EMG *t* tests for Monkey N (9, 19, 8, 6); and Monkey L (6, 7, 13, 14). Error bars indicate mean and SE.

Figure 6 shows how both the original and normalized MEPs changed from the pretraining to the post-training recordings made on the same day. The only statistically significant effect observed between pretraining and post-training sessions was a reduction in M1 MEP size in Monkey N (Fig. 6*A*); however, this was lost with normalization by background EMG (Fig. 6*B*) and was not seen in Monkey L.

Increasing load in the strength training sessions was associated with a reduction in background EMG activity in Monkey N but had no such effects in Monkey L, in the post-training session compared with the pretraining session (Fig. 6*C*). This variation in background EMG activity provides justification for the MEP normalization method previously described.

### Long-term training adaptations

**Figure 7.**
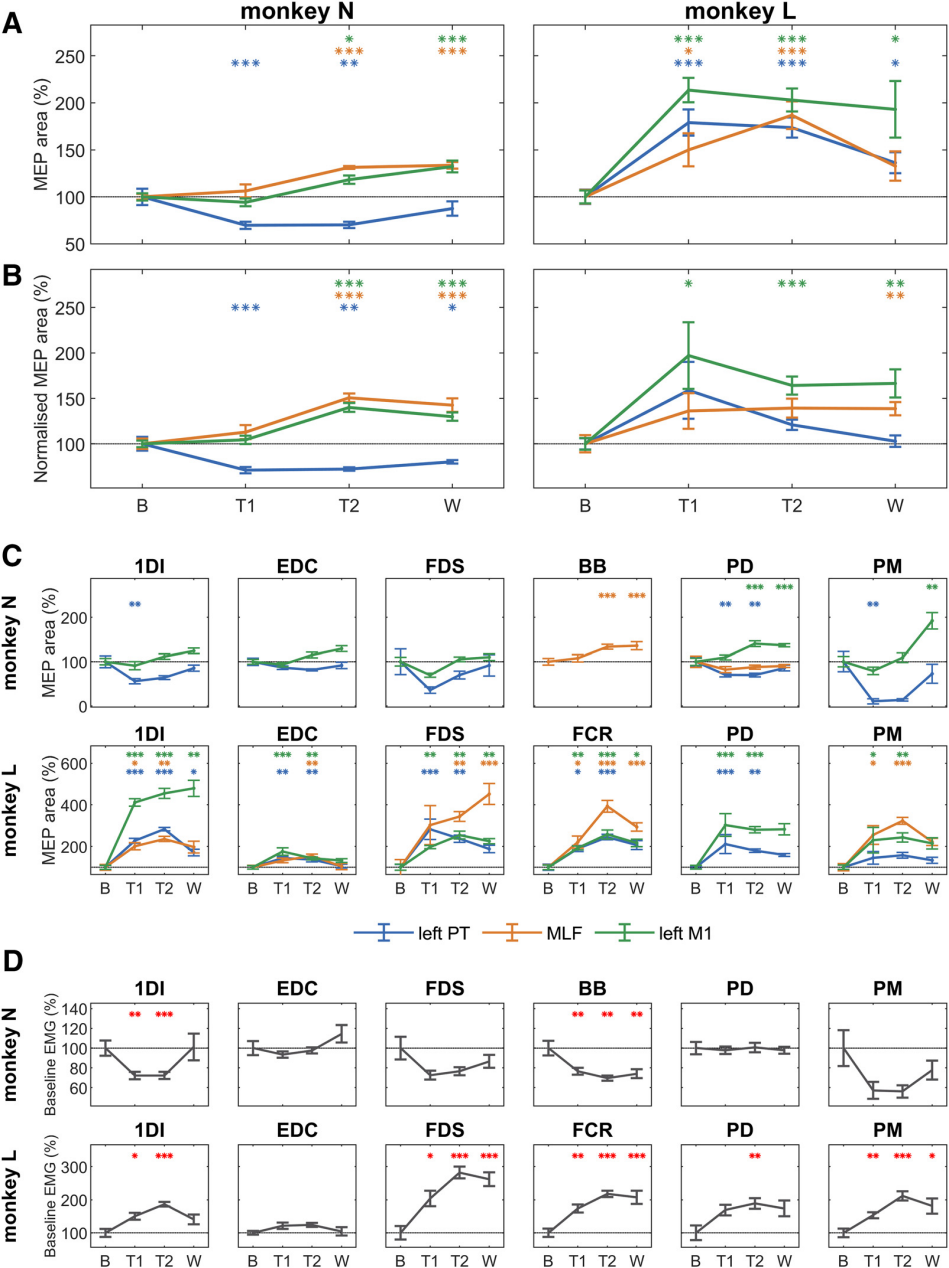
Long-term adaptations to strength training in the right (trained) arm. Change in MEP size recorded from muscles on the right (trained) arm relative to the baseline period. MEP area was calculated as the area under the curve above background EMG activity for a custom window for each muscle-stimulus combination. MEP size in the training 1 (T1), training 2 (T2), and the washout (W) periods was compared with MEP size in the baseline (***B***) period with independent two-tailed *t* tests and multiple comparisons corrected within each monkey using a Benjamini-Hochberg correction with a false discovery rate of 5%. Statistically significant change (*p < 0.05; **p < 0.01; ***p < 0.001) in MEP size relative to the baseline (***B***) period. ***A***, Change in MEP size averaged across all included muscles following inverse-variance weighting of individual muscle percentages. Degrees of freedom (T1, T2, W) for Monkey N: left PT (28.0, 11.9, 17.0), MLF (25.0, 29.0, 17.0), and left M1 (29.0, 28.0, 17.0); and Monkey L: left PT (23.9, 23.9, 13.0), MLF (22.7, 24.7, 13.0), and left M1 (20.6, 25.0, 7.7). ***B***, Same, but with normalization of values relative to background EMG. Degrees of freedom (T1, T2, W) for Monkey N: left PT (28.0, 10.1, 10.1), MLF (28.0, 29.0, 17.0), and left M1 (29.0, 28.0, 17.0) and Monkey L: left PT (19.3, 25.0, 13.0), MLF (23.6, 25.0, 13.0), and left M1 (15.9, 24.5, 9.4). ***C***, Percentage change in MEP size for individual muscles. Degrees of freedom (T1, T2, W) for Monkey N: IDI-left PT (28.0, 29.0, 17.0), IDI-left M1 (29.0, 28.0, 17.0), EDC-left PT (28.0, 10.1, 17.0), EDC-left M1 (29.0, 28.0, 17.0), FDS-left PT (10.0, 11.9, 17.0), FDS-left M1 (10.0, 11.7, 12.5), BB-MLF (25.5, 29.0, 17.0), PD-left PT (28.0, 29.0, 17.0), PD-MLF (26.0, 11.2, 17.0), PD-left M1 (29.0, 28.0, 11.4), PM-left PT (9.3, 9.2, 17.0), and PM-left M1 (29.0, 28.0, 17.0). Degrees of freedom (T1, T2, W) for Monkey L: IDI-left PT (22.8, 25.0, 13.0), IDI-MLF (23.0, 25.0, 13.0), IDI-left M1 (18.1, 22.7, 7.4), EDC-left PT (23.8, 24.6, 13.0), EDC-MLF (21.1, 22.7, 8.5), EDC-left M1 (20.7, 25.0, 8.4), FDS-left PT (23.5, 25.0, 13.0), FDS-MLF (19.4, 19.9, 11.0), FDS-left M1 (20.0, 25.0, 6.6), FCR-left PT (21.0, 23.0, 9.5), FCR-MLF (21.9, 24.2, 13.0), FCR-left M1 (20.0, 25.0, 13.0), PD-left PT (21.4, 23.2, 8.6), PD-left M1 (21.0, 22.9, 7.7), PM-left PT (24.0, 25.0, 13.0), PM-MLF (24.0, 24.0, 12.0), and PM-left M1 (19.2, 24.9, 9.4). ***D***, Change in background EMG activity recorded from muscles on the right (trained) arm relative to the baseline period. Background EMG was calculated as mean rectified EMG activity measured over a 40 ms window (−50 to −10 ms) before each stimulus. Asterisks indicate a statistically significant change (*p* < 0.05) in background EMG relative to the baseline period, as described above. Degrees of freedom (T1, T2, W) for Monkey N: IDI (30.0, 30.0, 17.0), EDC (30.0, 30.0, 17.0), FDS (11.8, 11.4, 17.0), BB (28.0, 11.5, 17.0), PD (30.0, 30.0, 17.0), and PM (30.0, 11.2, 17.0); and Monkey L: IDI (23.0, 25.0, 13.0), EDC (24.0, 25.0, 13.0), FDS (24.0, 25.0, 13.0), FCR (24.0, 25.0, 13.0), PD (24.0, 25.0, 13.0), and PM (24.0, 25.0, 13.0). Error bars indicate mean and SE.

In order to measure long-term changes in outputs induced by the strength training program, we measured the MEPs in the pretraining sessions on each day. Figure 7*A* presents the results for the raw MEP sizes, uncorrected for background EMG changes. As these could have been affected by the background EMG changes shown in Figure 7*D*, Figure 7*B* provides an alternative presentation of MEP values normalized to background. Similar trends were observed in both datasets. Both monkeys showed a significant facilitation of M1 MEPs. The MLF MEPs also increased in amplitude in both animals. There was no consistent trend for PT MEPs, which showed a significant suppression in Monkey N and no change in Monkey L (Fig. 7*B*). Results for individual muscles are shown in Figure 7*C* (MEPs) and Figure 7*D* (background EMG).

### Spinal adaptations

**Figure 8.**
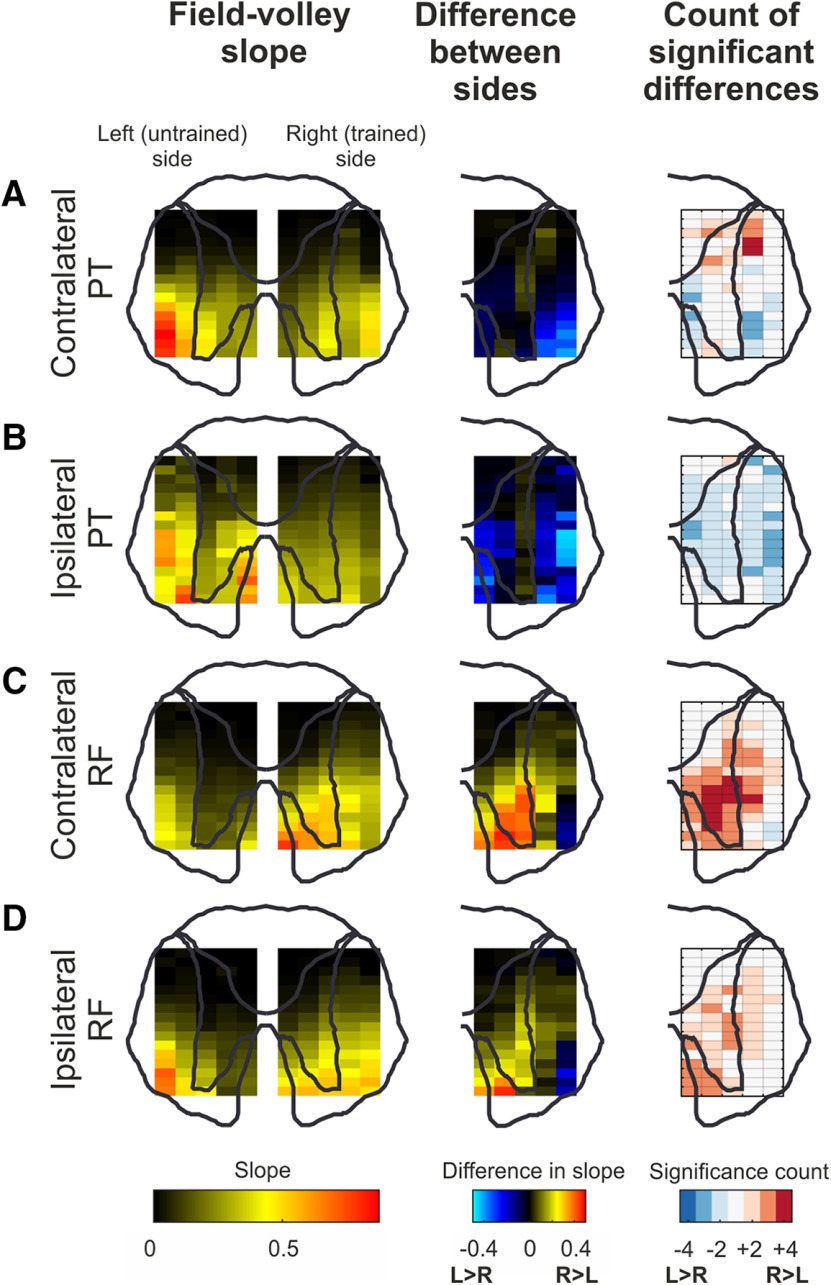
Spinal adaptations to strength training. Field-volley gradients are presented in the first column for contralateral PT volleys (***A***), contralateral RF volleys (***C***), ipsilateral PT volleys (***B***), and ipsilateral RF volleys (***D***). PT and RF volleys are measured from the areas corresponding to DLF and VLF, respectively (see Fig. 2*F*). Outline of the cord indicates the approximate location of each measurement. Second column represents the difference in gradient between the left and right side of the cord for each stimulus. Third column represents the statistical significance of this gradient difference (see Materials and Methods; Fig. 2*G–I*).

Figure 8 presents maps of spinal response gain, calculated as described in Materials and Methods. Each row illustrates data from a different stimulus location (PT or RF) and side (ipsilateral or contralateral to the spinal recording site). The left column shows a normalized map of gain, averaged across the four available recordings (two per monkey, in 2 animals). The middle column illustrates a difference map between the two sides. Finally, the right column shows a count, across the four available recordings, of the excess of sites with a significant different between the two sides in either direction; this has been thresholded, so that white boxes represent sites with no significant effect above-chance levels.

Within the gray matter, there were few significant differences between the gain on each side in response to contralateral PT stimulation (Fig. 8*A*). There was, however, a cluster of significant points in the white matter, in the region of the VLF, with a smaller field in this region on the trained side than on the untrained side. A similar result was seen following ipsilateral PT stimulation (Fig. 8*B*), although now a diffuse significant effect was seen over much of the cord, with the trained side showing a smaller response than the untrained side.

In contrast, the spinal gain in response to contralateral RF stimulation was significantly greater in the ventral horn and intermediate zone on the right (trained) side; this was often consistent in all four recordings (Fig. 8*C*, right, dark red). The gain following ipsilateral RF stimulation showed less consistent changes, although there was still a significant increase of trained versus untrained side over much of the ventral and intermediate gray matter (Fig. 8*D*).

## Discussion

**Figure 9.**
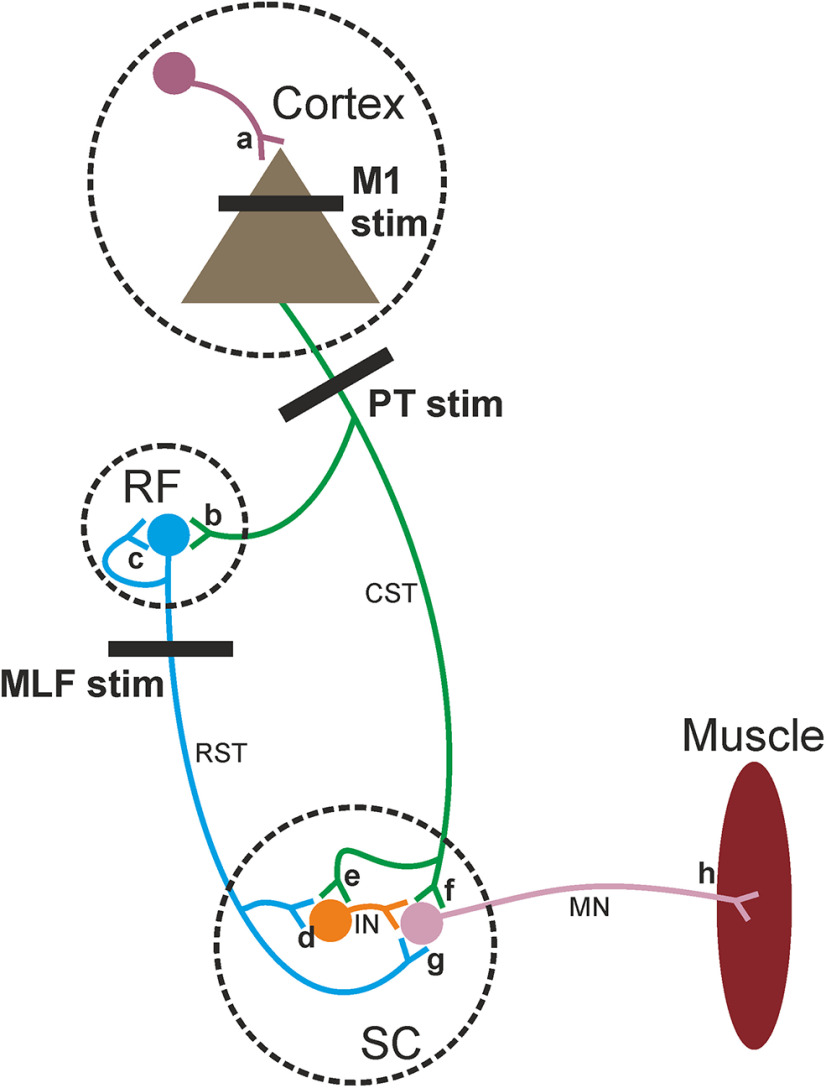
Schematic showing simplified pathways. Strength training may induce adaptive changes in the following: (***a***) intracortical circuits, (***b***) corticoreticular connections, (***c***) reciprocal reticular connections, (***d***) reticulospinal projections to interneurons, (***e***) corticospinal projections to interneurons, (***f***) corticomotoneuronal synapses, (***g***) monosynaptic reticular projections to motoneurons, and/or (***h***) within the motor units themselves.

The human strength training literature has used noninvasive techniques to investigate the neural changes associated with strength gains. Studies have predominantly focused on TMS to assess cortical changes and reflex measures to examine spinal adaptations. Noninvasive techniques to measure reticulospinal output directly in humans are not currently available. In this study, we used invasive measures in awake behaving monkeys to assess reticulospinal function as well as intracortical and corticospinal circuitry. Figure 9 presents a schematic illustration of the relevant neural connections, and potential sites for adaptations to occur, which will be referred to throughout the Discussion.

### Cortical and corticospinal contributions

The observed facilitation of M1 MEPs in the absence of a similar trend in PT MEPs suggests that neural adaptations occur at the cortical level (Fig. 9*a*) with strength training. This is consistent with much of the human literature. A recent meta-analysis reported a large effect of strength training interventions for decreasing short-interval intracortical inhibition and a medium effect on reducing silent period duration (Kidgell et al., 2017), suggesting an overall effect of reducing cortical inhibition.

The facilitation of M1 MEPs without a corresponding trend in PT MEPs also excludes the possibility that adaptations occurred at the cortico-motoneuronal synapse (Fig. 9*f*). In addition to our inconsistent MEP findings, we did not observe any clear side-to-side differences in PT-elicited responses in parts of the spinal cord corresponding to the intermediate zone or motor nuclei. This suggests that either a bilateral adaptation has occurred, or that strength training does not have a significant effect on corticospinal synapses. We cannot draw conclusions about the disynaptic action of the CST on motoneurons (Fig. 9*e*) since this pathways is rarely activated by PT stimulation without attenuation of feedforward glycinergic inhibition (Maier et al., 1997, 1998; Alstermark et al., 1999; Isa et al., 2006).

### Reticulospinal contributions

We are not aware of any previous reports of reticulospinal adaptations with strength training. Our finding of a facilitation of MLF MEPs is therefore novel but perhaps not surprising. Following bilateral PT lesions in monkey, Lawrence and Kuypers (1968) commented that “The most striking change after the first four to six post-operative weeks was a progressive increase in their general strength.” Given the absence of corticospinal projections in these animals, this increase in strength must have had an extrapyramidal substrate. Subsequent work has directly implicated the RST in this recovery process by showing that reticulospinal projections can strengthen following corticospinal lesions (Zaaimi et al., 2012), and that cells within the RF increase their firing rate (Zaaimi et al., 2018b). Furthermore, a recent study proposed that the RST and CST may constitute two separable systems for recovery following stroke, with the RST mostly contributing to strength (Xu et al., 2017).

The extensive collateralization of the RST (Peterson et al., 1975; Matsuyama et al., 1997) enables activation of muscle synergies. This is compatible with a role in strength training, which typically involves gross movements requiring coactivation of several muscles. Our simple lever-pulling task generated substantial EMG activity in all recorded muscles on the active arm (Fig. 3), thus showing more similarity to the gross movements of the RST (Davidson and Buford, 2004, 2006) than the sophisticated individuation associated with corticospinal function (Zaaimi et al., 2018a).

We assessed reticulospinal function through MLF stimulation in awake behaving monkeys. The observed facilitation of MLF MEPs suggests an increase in the synaptic efficacy of reticulospinal inputs to the spinal cord. In support of this, after a further 3 months of strength training, spinal circuits demonstrated a greater output for a given RST input on the trained compared with the untrained side. Our method cannot provide quantification of absolute changes in synaptic efficacy, instead simply providing a comparison between the two sides of the cord. It is thus possible that the response to RST inputs was enhanced bilaterally, but that this effect was greater on the trained side. Such an interpretation would be consistent with the cross-education literature: the untrained side does become stronger after unilateral training, but to a lesser extent than the trained side. Individual RST axons project bilaterally to the cord; our results showing greater increases in RST input to the trained side suggest that terminals from the same axon may have been affected differently based on their postsynaptic contacts.

The RST forms both monosynaptic and disynaptic connections with upper limb motoneurons (Riddle et al., 2009). The increased synaptic efficacy in the right (trained) cord appeared in both the intermediate zone and the motor nuclei (Fig. 8*C*). This suggests that changes in reticulospinal output following strength training occur both at reticulo-interneuron (Fig. 9*d*) and reticulo-motoneuron synapses (Fig. 9*g*).

We observed side-to-side differences in output gain not only in the gray matter, but also extending to the VLF. There was a decrease in gain in this region following PT stimulation, and an increase following RF stimulation, independent of which side was stimulated (Fig. 8). Stimulus trains delivered to the PT or RF produce a later, supernumerary volley thought to represent indirect (transsynaptic) activation of reticulospinal cells by collaterals of the stimulated corticospinal or reticulospinal axons (Jankowska et al., 2003; Edgley et al., 2004; Fisher et al., 2015). This is in some ways analogous to the indirect waves of corticospinal output produced following cortical stimulation (Rosenthal et al., 1967; Di Lazzaro et al., 2004). The potentials measured as field within the VLF are most likely this supernumerary volley. The differences seen between sides in the gain of this potential therefore probably reflect changes in synaptic efficacy caused by the strength training within the RF, and not at a spinal level. This suggests that strength training produces a decrease in cortico-reticular connections (Fig. 9*b*), but an increase in reticular-reticular connectivity (Fig. 9*c*).

We reject the hypothesis that the observed adaptations are entirely due to postsynaptic changes in either motoneurons or interneurons, since many of these receive convergent reticulospinal and corticospinal inputs (Riddle et al., 2009; Riddle and Baker, 2010). If postsynaptic adaptations were a dominant effect, we would expect to see similar trends for reticular and corticospinal stimuli, which was not the case. Although changes in motoneuron properties were observed in rodents with strength training (Krutki et al., 2017), the differences between the MEPs observed with PT, MLF, and M1 stimulation in our experiments suggest that motoneuron changes are not the dominant factor. In theory, increased motoneuron excitability combined with decreased PT efficacy, in the absence of any MLF and M1 changes, could explain some of our findings, but this is unlikely, especially in the context of the results from the spinal recordings.

In conclusion, strength training likely generates neural adaptations throughout the motor system, both unilaterally and bilaterally. We propose that, for gross upper body movements, these adaptations primarily occur in intracortical and reticulospinal networks. The latter likely consists of changes in synaptic efficacy between descending reticulospinal projections and either motoneurons or interneurons, as well as possible changes within the RF itself. Our results suggest that neither motoneuronal nor corticospinal adaptations play a major role. These findings highlight reticulospinal pathways as deserving new attention in the strength training field.
